# Comparative Mortality of Typhoid Fever in Kentucky and Missouri

**Published:** 1849-03

**Authors:** S. W. Southworth

**Affiliations:** Glasgow, Missouri


Art. VII.—*Comparative Mortality of Typhoid Fever in Kentucky and Missouri.* In a letter to the Editor, by S. W. Southworth, M.D., of Glasgow, Missouri.

Since the great overflow in 1844, we have not had a healthy season in this part of Missouri until the present one. But little disease has prevailed since last winter, when typhoid fever was prevalent. I have been struck by the fact, which I deem worthy of the notice of the medical philosopher, that this fever is so much more fatal in Kentucky than in Missouri. In my practice, which is an extensive one, I have not lost, on an average, more than two cases a year from this fever, while I find that in the neighborhood of my old home, in Woodford county, Ky., some families have lost in the same period ten or twelve members. This shows a striking difference in the malignity of the disease in the two regions. The question is, what leads to the difference? That is reputed a healthy region, while this is eminently miasmatic. That has been long settled and is in a high state of cultivation; this is comparatively a new country, and much of it is still covered by its primeval forests. Can this be the cause of the diversity? Can it be that a miasmatic influence neutralizes, to some extent, the poison of the typhoid disease, or that the condition of the system engendered by it does not allow of as violent febrile action? In nearly all our cases, we find engorgement of the liver or spleen as among the symptoms, indicating the extent to which the malarious influence prevails. What connection there is, or whether any, between this condition of our climate and the comparatively mild character of typhoid fever, I am not prepared to say, but the fact strikes me as interesting, and as such I communicate it to you.

November, 1848.

